# P02.12. Temporal dynamics of symptom and treatment variables in a lifestyle-oriented approach to anxiety disorder: a single-subject time-series analysis

**DOI:** 10.1186/1472-6882-12-S1-P68

**Published:** 2012-06-12

**Authors:** R Hoenders, E Bos, P de Jonge

**Affiliations:** 1 Center for Integrative Psychiatry, Lentis, Groningen, Netherlands; 2 Interdisciplinary Center for Psychiatric Epidemiology, RuG, UMCG, Groningen, Netherlands

## Purpose

Although there is increasing evidence for the positive effects of a healthy lifestyle on mental health, most studies only take into account a single lifestyle factor, ignore the possibility of bidirectional causality, and focus on average group results.

## Methods

In the present single-subject study, we used multivariate time-series analysis (Vector Auto Regressive modeling) to unravel the dynamic interplay between symptom and treatment variables in a multi-component treatment of anxiety disorder. Main treatment variables were two lifestyle factors (physical activity and relaxation).

## Results

The patient in this study recovered completely. Time-series analysis revealed an intricate pattern of dynamic relationships between symptom and treatment variables. Relaxation was predictive of symptom reduction but physical activity surprisingly worsened the symptoms. Changes in energy predicted changes in anxiety. Evidence for bidirectional causality was present as well, with changes in relaxation predicting changes in energy and vice versa, indicating a positive feedback loop.

## Conclusion

This study aimed to unravel the dynamic relationships between psychological symptoms and the health-related behaviors intended to improve these symptoms. These relationships turned out to be characterized by bidirectionality, lagged influences, indirect effects, and feedback loops, both between symptoms and behaviors as well as among them. This patient’s symptoms and behavior were interrelated in an intricate way. This type of research seems useful for gaining insight into the causal mechanisms underlying the effects of a healthy lifestyle on mental health.

